# Novel *PAK3* gene missense variant associated with two Chinese siblings with intellectual disability: a case report

**DOI:** 10.1186/s12881-020-0957-x

**Published:** 2020-02-12

**Authors:** Yanyan Qian, Bingbing Wu, Yulan Lu, Wenhao Zhou, Sujuan Wang, Huijun Wang

**Affiliations:** 1 Center for Molecular Medicine, Children’s Hospital of Fudan University, Shanghai Key Laboratory of Birth Defects, Shanghai, 201102 China; 2 Departments of Rehabilitation, Children’s Hospital of Fudan University, Shanghai, 201102 China; 3 Pediatrics Research Institute, Children’s Hospital of Fudan University, 399 Wanyuan Road, Shanghai, 201102 China

**Keywords:** Intellectual disability (ID), Exome sequencing, *PAK3*, Pathogenic variants

## Abstract

**Background:**

Intellectual disability (ID) constitutes the most common group of neurodevelopmental disorders. Exome sequencing has enabled the discovery of genetic mutations responsible for a wide range of ID disorders.

**Case presentation:**

In this study, we reported on two male siblings, aged 4 and 2 years, with motor and mental developmental delays and mild dysmorphic facial features. To identify the genetic causes of these symptoms, we employed trio-whole exome sequencing for the proband. We found a novel hemizygous missense variant in the *PAK3* gene (c.1112G > A, p.Cys371Tyr), which encodes the p21-activated kinase 3, in the proband, which inherited from mother. The younger brother also has the hemizygous variant, which was confirmed by Sanger sequencing. The variant is located in the kinase domain and was regarded as a likely pathogenic variant in this family.

**Conclusion:**

We diagnosed two male siblings with developmental delays as having a *PAK3* likely pathogenic variant. This finding expands the list of *PAK3* gene mutations associated with neurodevelopmental disorders and provides further details on its clinical features.

## Background

Intellectual disability (ID) is the terminology used to describe the significant impairment of cognitive and adaptive development due to abnormalities in brain structure or function. ID is a common clinical feature in pediatrics. Early identification and intervention are essential for affected patients. Genetic causes of ID are present in 25–50% of clinical cases [1]. The extensive use of next-generation sequencing (NGS) has resulted in the identification of genetic findings in increasing numbers of patients with developmental disorders [2].

The *PAK3* (P21 Activated Kinase 3) gene, which encodes a protein containing a kinase domain (KD) and a p21-binding domain/autoinhibitory domain (PBD/AID), is associated with X-linked non-specific intellectual disability and is known to cause non-syndromic mental retardation X-linked 30/47 (MIM 300558). In the Pak3-knockout mice, oligodendrocyte differentiation in the corpus callosum and the process of synaptic plasticity were affected, the mice showed learning and memory deficiency [3, 4].

To date, there have been 15 families comprising 47 patients reported with PAK3 mutations. The patients showed variable face features and psychiatric manifestations, and all had mild to severe ID. In this study, we identified one novel hemizygous missensevariant of *PAK3* in two siblings in one family. We also reviewed the previous cases of *PAK3* defects and summarized the phenotype and genotype correlation.

## Case presentation

### Clinical presentation and family history

**Fig. 1:**
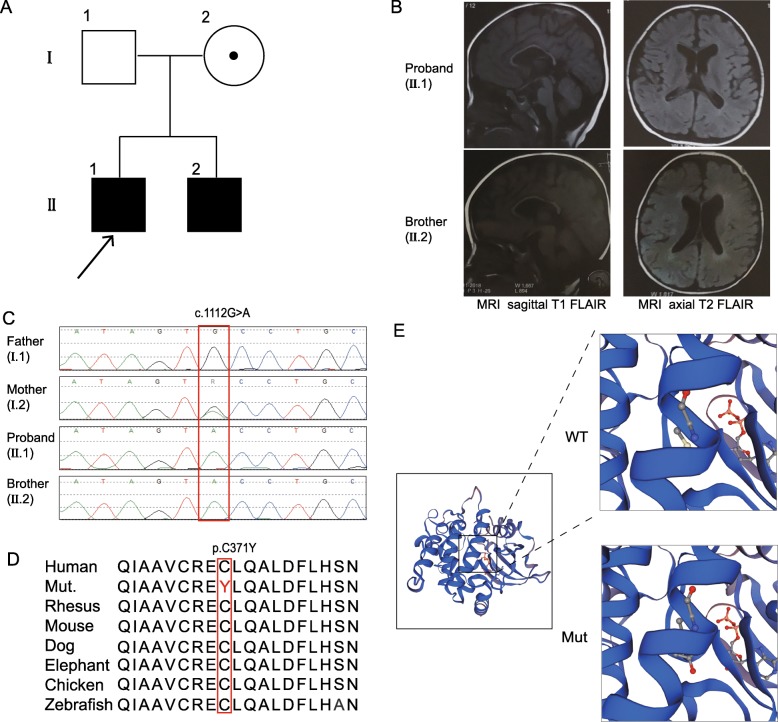
The novel likely pathogenic variant of the *PAK3* gene and the brain MRI scans of the patients. **a** The family tree. **b** The left sides show sagittal T1 FLAIR images with widened lateral ventricles and thin corpus callosum. The right sides show axial T2 FLAIR images with enlarged lateral ventricles. **c** The hemizygous variant detected by whole-exome sequencing and confirmed by Sanger sequencing. **d** The variant region is conserved among human, rhesus, mouse, dog, elephant, chicken and zebrafish. **e** The predicted structure of the mutated PAK3, with the site of the mutation in the enlarged views

The proband was a 3-year-10-month-old boy at the time of admission (Fig. 1A). He was born to a nonconsanguineous couple by normal delivery at 42^+ 2^ weeks. His birth weight was 3200 g (− 0.2SD), and his Apgar score was 10. He did not meet the developmental milestones and was therefore brought to visit a doctor. He raised his head at 3 months, turned over at 6^+^ months, and sat up at 9 months. He was able to grab a toy with his hands by himself at 1 year old. He could crawl on his hands and legs and walk independently at 2 years old. He could pinch things with thumb and index finger at 3 years of age but had difficulty putting down objects or releasing them from his fingers. He recognized people at 2.5 years, and could say a single word at 3-year-10-month-old. In the last follow up, he could say a simple sentence at 4-year-2-month-old but could not read or write. However, the acquired skills were not retrogressed. He liked to push people and seemed aggressive. He did not present with seizures but rubbed his legs with his hands while clenching his teeth and demonstrating esotropia for approximately 1 min. This behavior could be interrupted. A 24-h sleep electroencephalogram (EEG) showed little abnormality at 4 years old. Head magnetic resonance imaging (MRI) showed lateral ventricle enlarged, white matter around the lateral ventricle decreased, and with corpus callosum dysplasia (Fig. 1B). His head circumference was 48.6 cm (− 1.2SD) at 4-year-2-month-old. The boy had mild dysmorphic facial feature (hypertelorism, slight epicanthus, esotropia, mild nasal bridge depression, oral hypotonia, open mouth, high palatal arch, large ears). He had a rash in infancy, and at the last follow-up he had psoriasis on the head, back, and shins and depigmentation spots on the forehead. His auditory and visual responses were normal.

The younger brother was a 2-year-1-month-old boy who was delivered at 42 weeks. His birth weight was 3800 g (+ 0.4SD). He raised his head at 3 months, turned over at 5 months, and sat up at 14 months. He could hold something with his hands by himself at 1 year old and could pinch something with the thumb and index finger until 1.5 years old. In addition, he understood commands at 1.5 years old. He could crawl on his stomach at 23 months, but he still could not crawl with his hands and legs or walk independently. He cannot build blocks, read or write but did not otherwise retrogress. He did not show any abnormal behaviors. The EEG was normal. The head MRI showed lateral ventricle enlarged, white matter around lateral ventricle decreased, and with corpus callosum dysplasia (Fig. 1B). His head circumference was 46.1 cm at 1-year old (− 1.6SD) which was relative small. He also had mild dysmorphic facial features, including hypertelorism, slight epicanthus, esotropia, nasal bridge depression, oral hypotonia, open mouth, high palatal arch, and large ears. He had rash when in infancy and had depigmentation spots on the forehead. Psoriasis appeared in his torso. His auditory and visual sense was normal.

The father and grandparents were healthy. The mother graduated from junior high school with slight difficulty; she had mild facial features, including open mouth and esotropia. The physician and her husband opined that she had a relatively rigid character, which indicating the mother have a mild phenotype.

### Genomic analysis

Genomic DNA of the children and their parents was extracted from whole blood using a QIAamp DNA Blood Mini Kit (Catalog no. 51106). Nucleic acid preparation and high-throughput sequencing were performed according to standard protocols in the Clinical Laboratory Improvement Amendments (CLIA) compliant sequencing laboratory in Wuxi NEXTCODE (288 Fute Zhong Road, Waigaoqiao Free Trade Zone Shanghai 200,131, China CLIA ID 99D2064856). Whole-exome capture was performed using an Agilent SureSelect Human All Exon 50 Mb Kit (Agilent Technologies, Santa Clara, California, USA) followed by sequencing as 150-bp paired-end runs on an Illumina XTen (Illumina, San Diego, CA, USA) platform. Segregation of the PAK3 variant within the family was confirmed by Sanger sequencing on the ABI 3730 Genetic Analyzer (Applied Biosystems, Foster City, CA, USA).

Sequence data were mapped to the human reference genome (GRCh37/hg19). Variants calling was using the Genome Analysis Toolkit Best Practices Pipeline (Version 3.2.2) [5]. Data filtering, variant prediction, and interpretation followed ACMG guidelines and those from our previous work [6, 7]. Variant analysis was based on the ExAC database (http://exac.broadinstitute.org/), the 1000 Genomes database (http://www.internationalgenome.org/) and an internal database (more than 30 k samples). The variants were predicted by the online software platforms PolyPhen2.2 (http://genetics.bwh.harvard.edu/pph2/), SIFT (http://sift.jcvi.org/), and MutationTaster (http://www.mutationtaster.org/). The structures of PAK3 and its mutant were analyzed using SWISSMODEL (https://www.swissmodel.expasy.org) base on the protein data bank (PDB ID: 6FD3). And the stability results of the wildtype and variant form of protein are reported by MOE.

**Fig. 2:**
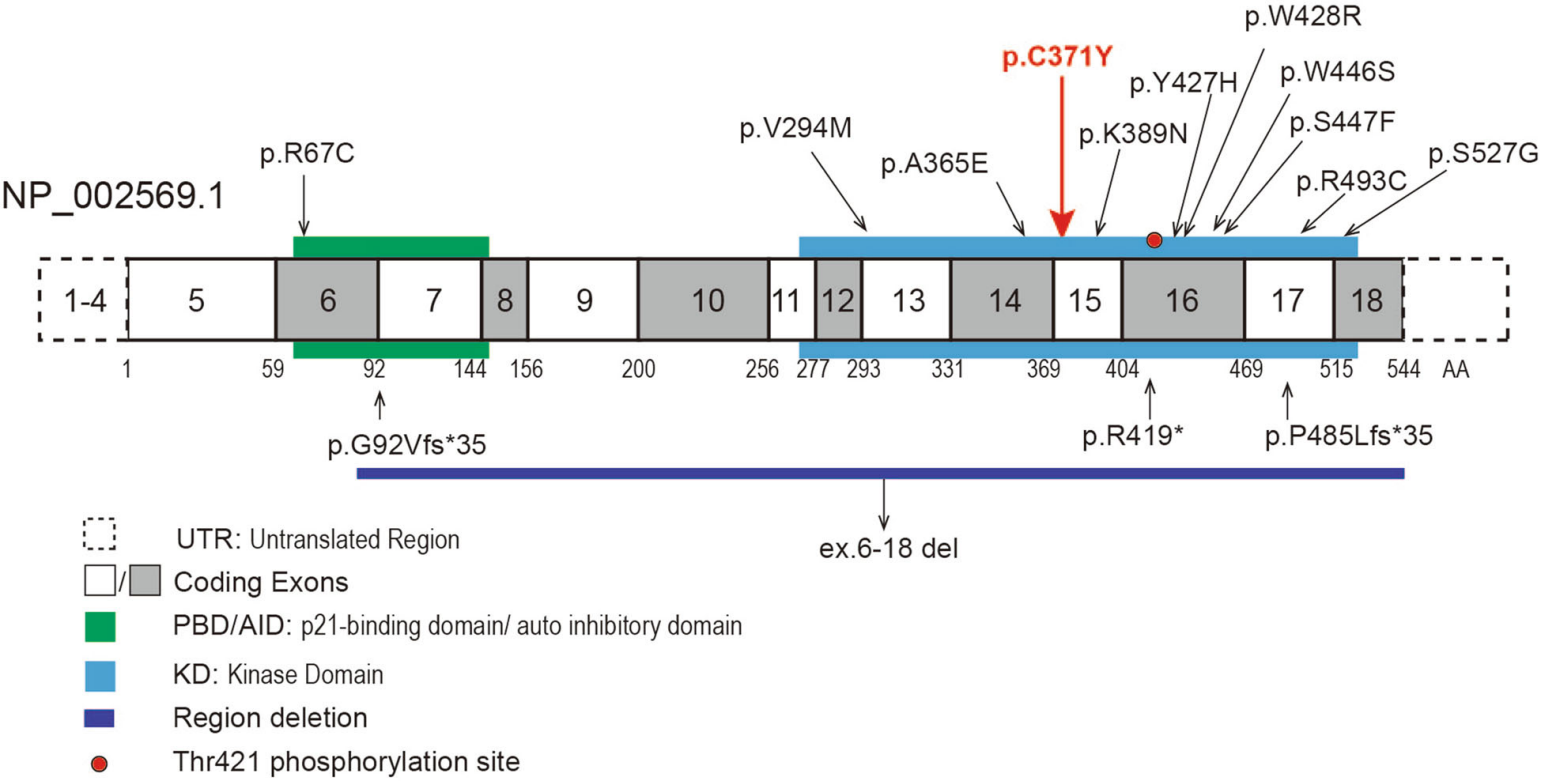
A schematic diagram of the mutation in PAK3. Boxes in gray/white with numbers are the exons. Square boxes with dotted lines are untranslated regions. The green frame represents the PBD/AID. The blue frame shows the KD. The dark blue strip shows the microdeletion. The red dot indicates the phosphorylation site at Thr421. The arrows point to the known mutation sites. The missense mutations are listed above the diagram, and the truncation mutations are listed below. The bold red arrow indicates the mutation detected in the present study

The mean depth of the trio-whole exome sequencing data was approximately 120×. We identified a novel variant (NM_ 002578: c.1112G > A, p.Cys371Tyr) of *PAK3* in the proband, which inherited from mother. Sanger sequencing confirmed his younger brother also had this hemizygous variant (Fig. 1C). The residue is conserved among species, including human, rat, mouse, dog, elephant, chicken, and zebrafish (Fig. 1D). The substituted tyrosine has one more benzene ring than cysteine, the senior structure of the variant protein was not obvious changed (Fig. 1E). The stability of the variant and wildtype protein was analysis using the Residue Scan module in MOE, showed a reduction of variant protein by 3.45 kcal/mol (Additional file 1: Table S1). The variant is located in the kinase domain of the protein (Fig. 2). The variant was not reported in the 1KG, the ExAC, the HGMD, or PubMed and is not recorded in our in-house database (approximately 30,000 samples). The potential pathogenicity of this variant was scored 0, 0.997 and 1 by SIFT, PolyPhen2, and MutationTaster, respectively, which means that the variant was classified as “damaged/disease-causing”. We concluded the variant as a likely pathogenic variant according to the ACMG guidelines, based on two affected patients in this family, the PP1 criteria can be upgraded to PM (Table 1). The other variants consisten with the inheritance model in this family were listed in Additional file 1: Table S2.

**Table 1:** The novel *PAK3* gene variant found in this study

Gene	Position	Variants	Zygosity	Inheritance	Frequency (ExAC/1KG/ InDa)	Prediction SIFT/PP/MT/CADD_phred	Variant classify following ACMG guideline
*PAK3*	chrX: 110439071	NM_ 002578: c.1112G > A; p.Cys371Tyr	Hemi	Maternal	0/0/0	D(0)/ D(0.997)/ D(1)/ 27.7	PM2 + PP1_PM + PP3 + PP4

*Annote* Hemi, Hemizygous, *InDa* inhouse database, *PP* polyphen 2, *MT* MutationTaster, *CADD* Combined Annotation Dependent Depletion

## Discussion and conclusion

Intellectual disability is a clinically homogeneous but genetically heterogeneous disorder. In 1998, Allen et al. first identified a point mutation (c.1255C > T, p.Arg419*) of the *PAK3* gene that affected individuals in a multiplex pedigree with X-linked ID. Including this study, 14 *PAK3* mutations in 15 families (family 8 and 9 share the same mutation) have been reported, including 11 missense, 1 nonsense, 1 splicing, and 1 frameshift mutation. Twelve of them were located in the KD, and two were in the PBD/AID. Currently, only 1 deletion in exon 6–18 has been reported, which covers the major area of the PBD/AID and all of the KD [2, 8–20] (Table 2, Fig. 2).

**Table 2:** The summary of genotype and clinical features of patients with *PAK3* mutations

family ID/ reference	family 1(this study)		family 2 Bienvenu T et al., 2000 [9]	family 3 Rejeb I,et al., 2008 [16]	family 4 Muthusamy B, et al., 2017, [11]	family 5 Gedeon AK, et al., 2003 [19]	family 6 Magini Pet al, 2014 [10]	family 7 Allen KM, et al., 1998 [12]	family 8
	Proband	Younger brother							Al-Shamsi A et al., 2016, [18]
NM_002578	c.1112G > A		c.199C > T	c.276 + 4A > G	c.880G > A	c.1094C > A	c.1167G > T	c.1255C > T	c.1279 T > C
NP_002569	p.Cys371Tyr		p.Arg67Cys	p.Gly92ValfsX35	p.Val294Met	p.Ala365GLu	p.Lys389Asn	p.Arg419*	p.Tyr427His
Domain	KD		PBD/AID	PBD/AID	KD	KD	KD	KD	KD
Cases sex (Age)	2 males (4y2m; 2y1m)		6 males (NA)	4 males (27y, 21y)	3 males (7y, 3y, 25y)	13 males (4y 3mo-69y)	2 males (2y 9mo, 6y)	4 males (NA)	1 male
Inheritance	Maternal		Maternal	Maternal	Maternal	Maternal	Maternal	Maternal	NA
Clinical phenotype									
Facial feature	+	+	–	+	+	+	+	NA	NA
Head MRI/ circumference abnormal	LVE, WMD, CCA/ relative small	LVW, CCA/ relative small	NA/ NA	NA/microcephaly	NA/ microcephaly	NA/−	CCA, LVE, CeH/ microcephaly	NA/ microcephaly	NA/ macrocephaly
EEG/Epilepsy	+/−	−/−	NA/−	- / -	NA/ -	NA/ -(1 patient +)	+/ +	NA/ -	NA/ NA
Motor development delay	+	+	+	+	+	+	+	+	+
Language development delay	+	+	+	+	+	+	+	+	+
Behavior abnormal	HyA, Agg	–	NA	Agg, HyA, et al	ADHD; Agg	Agg & antisocial, et al	–	1 patient, HyA, et al	NA
Other abnormals	rash infancy and pigment loss spots in the forehead in both siblings, psoriasis in older brother		–	hypotonia	pes planus; hypogenitalism	–	Severe ichthyosis, pectus excavatum, camptodactyly and syndactyly; hypotonia	–	obesity
family ID	family 9 Hertecant J et al., 2017 [8]	Family 10 Iida A et al., 2019 [15]	family 11 Peippo M et al., 2007 [13]	family 12 Deciphering Developmental Disorders S, 2015 [2]	family 13 Deciphering Developmental Disorders S, 2015 [2]	family 14 McMichael G et al., 2015 [20]	family 15 Horvath GA et al., 2018 [14]	family 16 Cartwright A et al., 2017 [17]	
NM_002578	c.1279 T > C	c.1282 T > A	c.1337G > C	c.1340C > T	c.1454delC	c.1477C > T	c.1579A > G	ex.6-18del	
NP_002569	p.Tyr427His	p.Trp428Arg	p.Trp446Ser	p.Ser447Phe	p.Pro485Leufs*35	p.Arg493Cys	p.Ser527Gly	/	
Domain	KD	KD	KD	KD	KD	KD	KD	/	
Cases sex (Age)	2 males (4y)	2 male (siblings) (7y, 1y)	5 males (6y-46y)	1 female (NA)	1 male (NA)	1 male (NA)	1 male (17.5y)	1 male (6y)	
Inheritance	De novo	Maternal	Maternal	De novo	Maternal	NA	Maternal	De novo	
Clinical phenotype									
Facial feature	–	NA	+	+	NA	NA	NA	+	
Head MRI/ circumference	−/ macrocephaly	CA and CCA/ microcephaly	1 patient hydrocephalus; 2 patients microcephaly	CCA; microcephaly	NA/ NA	InVH/ NA	LVE, CCA, WMC/ -	NA/ relative small	
EEG/Epilepsy	NA/ -	NA/ 1 patient +	4 patients +/ 1 patients +	NA/ NA	NA/ NA	NA/ +	+/ +	NA/ NA	
Motor development delay	+	+	+	+	+	+	+	+	
Language development delay	+	+	+	NA	NA	+	+	+	
Behavior abnormal	Likely ASD, temper tantrums	autistic stereotype movement	Agg, HyA, psychosis, et al	Stereotypic behavior	NA	NA	Agg, restless, self-injury	ASD	
Other abnormals	hypotonia	Hypotonia	Asthma, respiratory infections; hypotonia, 3 mothers mild feature	Hypotonia, lower limb spasticity	eye movement abnormal	prematurely born; cerebral palsy	NA	myopia and astigmatism	

*Annote* ADHD: Attention deficit hyperactivity disorder, *Agg* aggression, *ASD/AF* autism disorder/ autistic features, *CCA* corpus callosum abnormal, *CeH* cerebellar hypoplasia, *CeA* cerebral atrophy, *HyA* hyperactivity, *InVH* intraventricular hemorrhage, *KD* kinase domain, LVD: lateral ventricle enlarged, *mo* month, *NA* not available, *PBD/AID* p21-binding domain/ auto inhibitory domain, *WMD* white matter decreased, *WMC* white matter cavitation; year; +: positive; −: negative

As the gene encoding p21-activated kinase 3, PAK3 is implicated in dendritic spine morphogenesis and is a key regulator of synaptic functions. The Ala365Glu and Arg419* completely abolish kinase activity [21]. These two variants, along with Lys389Asn and Trp446Ser, result in a failure to phosphorylate myelin basic protein when it is stimulated by the constitutively active form of Cdc42 [10]. Patients with these four mutations showed gross and fine motor skill delay, no or poor/inarticulate speech, epileptic seizures in some cases, and aggressiveness, hyperactivity or other psychoses [10, 12, 13, 19] (Table 2). Arg67Cys, located in the PBD/AID, results in a drastically decreased binding of PAK3 to the small GTPase Cdc42 and impaires the subsequent activation of PAK3 by this GTPase [21]. This mutation was identified in six male patients in one family. Patients in this family showed moderate to severe ID but without seizures, stature growth deficiency or abnormal facial features [9]. The mutations in the PBD/AID might decrease the binding of Cdc42 directly and disturb the pathway, while the mutations in the KD might decrease kinase activity. Therefore, mutations in both the KD and PBD/AID are associated with clinical ID through different molecular mechanisms. In addition, splicing (c.276 + 4A > G, p.Gly92Valfs*35) and deletion ex.6-18del both result in the loss of the PBD/AID and KD. Two patients with these mutations showed facial dysmorphism, microcephaly, and mild to severe ID [16, 17]. The novel likely pathogenic variant of Cys371Tyr identified in our study was located in KD. We performed the structural modeling of the variant protein, found no obvious changed from the wildtype protein. However, the stability of the variant protein calculated by MOE showed reduced. In summary, all patients in 16 families with *PAK3* mutations/deletions presented with mild to severe ID regardless of the domain in which the mutation was located.

The patient from family 15, a 17.5-year-old boy with a Ser527Gly mutation, showed severe self-injury and abnormal behaviors. He was treated with low-dose L-dopa/carbidopa and 5-hydroxytryptophan, which significantly improved his self-injurious behavior [14]. In this study, the symptoms of the proband were similar to the patient in family 15. Except for the dysmorphic face and ID, the proband also showed some aggression and hyperactivity. We provided the treatment information of patient in family 15 to clinician, and suggested that earlier treatment may improve the boy’s behavior and prevent the occurrence of severe self-injurious behavior.

The mother in this study showed a slight phenotype. Similarly, the female carrier in family 11 with the heterozygous mutation Trp446Ser showed mild ID and mild dysmorphic facial features, exotropia, hyperactivity, and short attention. Additional, one female patient was reported in family 12, the author descripted the patient had microcephaly, global developmental delay, hypotonia, corpus callosum dysplasia, and stereotypic behavior, who carried a de novo variant of Ser447Phe [2]. Therefore, females might have variable clinical features, for which skewed X-inactivation needs consideration in genetic counseling [13].

In conclusion, we first describe a novel missense variant of *PAK3* in two Chinese male siblings with ID and dysmorphic facial features. Additionally, trio-WES could be a useful diagnostic method for allowing timely treatment and genetic counseling.

## Supplementary information

**Additional file 1 Table S1**. Stability results of PAK3 and variant, produced by Molecular Operating Environment (MOE). **Table S2**. The summary of variants consistent with the inheritance model other than the PAK3 gene in this family from the trio-WES data.

## Data Availability

The likely pathogenic variant of the *PAK3* gene has been submitted to ClinVar (https://www.ncbi.nlm.nih.gov/clinvar/) with the accession number: SCV000986694. The data that support the findings of this study are available on request from the corresponding author. The raw data are not publicly available due to ethical restrictions.

## Abbreviations

ID: intellectual disability
KD: kinase domain
NGS: next-generation sequencing
PBD/AID: p21-binding domain/autoinhibitory domain
