# Supplementary material for: Novel PAK3 gene missense variant associated with two Chinese siblings with intellectual disability: a case report
Source: BMC Med Genet. 2020 Feb 12;21:31. doi: 10.1186/s12881-020-0957-x (PMC7017536; doi:10.1186/s12881-020-0957-x)
Supplement: Supplementary file 1 — Additional file 1 Table S1. Stability results of PAK3 and variant, produced by Molecular Operating Environment (MOE). Table S2. The summary of variants consistent with the inheritance model other than the PAK3 gene in this family from the trio-WES data. [file 12881_2020_957_MOESM1_ESM.docx]

Supplementary Table 1. Stability results of PAK3 and variant, produced by Molecular Operating Environment (MOE).

| PDB | Variant | Stability (kcal/mol) | 𝜟Stability(kcal/mol) |
| --- | --- | --- | --- |
| 6FD3 | C386C | -1132.21 | 0 |
| 6FD3 | C386Y | -1128.76 | 3.45 |

Supplementary Table 2. The summary of variants consistent with the inheritance model other than the PAK3 gene in this family from the trio-WES data

| **Gene** | **location** | **Variant** | **Zygo** | **OMIM** | **Inherit** | **Source** | **Annote** |
| --- | --- | --- | --- | --- | --- | --- | --- |
| *CEP290* | chr12:  88452656 | NM_025114:exon49:  c.6787A>G(p.S2263G) | Het | Joubert syndrome 5, [MIM:610188]; Leber congenital amaurosis 10, [MIM:611755]; Meckel syndrome 4, [MIM:611134]; Senior-Loken syndrome 6, [MIM:610189] | AR | Maternal | Phenotype mismatch |
| *CEP290* | chr12:  88480253 | NM_025114:exon33:  c.4217C>T(p.A1406V) | Het |  | AR | Paternal | Phenotype mismatch |
| *PDE4DIP* | chr1:  144874752 | NM_001198834:exon30:  c.4856C>T(p.A1619V) | Het | . | . | Maternal | Phenotype mismatch |
| *PDE4DIP* | chr1:  144882471 | NM_001198834:exon24:  c.3548C>T(p.A1183V) | Het | . | . | Paternal | Phenotype mismatch |
| *TTN* | chr2:  179455151 | NM_001267550:exon304:  c.61301T>G(p.V20434G) | Het | Cardiomyopathy, dilated, 1G, [MIM:604145]; Cardiomyopathy, familial hypertrophic, 9, [MIM:613765]; Muscular dystrophy, limb-girdle, type 2J, [MIM:608807]; Myopathy, proximal, with early respiratory muscle involvement, [MIM:603689]; Salih myopathy, [MIM:611705]; Tibial muscular dystrophy, tardive, [MIM:600334] | AD/AR | Maternal | Phenotype mismatch |
| *TTN* | chr2:  179455350 | NM_001267550:exon304:  c.61102C>A(p.P20368T) | Het |  | AD/AR | Paternal | Phenotype mismatch |
